# Heat shock proteins in the therapy of autoimmune diseases: too simple to be true?

**DOI:** 10.1007/s12192-019-01000-3

**Published:** 2019-05-09

**Authors:** Stefan Tukaj, Maciej Kaminski

**Affiliations:** 1 Department of Molecular Biology, Faculty of Biology, University of Gdańsk, Wita Stwosza 59, 80-308 Gdańsk, Poland; 2 Department of Anaesthesiology and Intensive Therapy, University Clinical Centre, Gdańsk, Poland

**Keywords:** Heat shock proteins, HSP, Autoimmune diseases therapy, Immunoregulation

## Abstract

Autoimmune diseases are characterized by the loss of immune tolerance to self-antigens which leads to an excessive immune responses and chronic inflammation. Although much progress has been made in revealing key players in pathophysiology of various autoimmune diseases, their therapy remains challenging and consists of conventional immunosuppressive treatments, including corticosteroids and more advanced biological therapies which are targeted at molecules involved in maintaining chronic inflammation. These therapies are focused on suppressing inflammation; nevertheless, a permanent balance between protective and pathogenic immune responses is not achieved. In addition, most of currently available therapies for autoimmune diseases induce severe side effects. Consequently, more effective and safer therapies are still required to control autoimmunity. Stress-induced cell protecting heat shock proteins (HSP) have been considered as a potential treatment targets for autoimmune diseases. HSP, predominantly intracellular components, might be released from bacteria or mammalian tissues and activate immune response. This activation may lead to either production of (auto)antibodies against HSP and/or induction of immune regulatory mechanisms, including expansion of desired T regulatory (Treg) cells. Because inadequate frequency or activity of Treg is a characteristic feature of autoimmune diseases, targeting this cell population is an important focus of immunotherapy approaches in autoimmunity.

Evolutionarily conserved heat shock proteins (HSP) are constitutively expressed and/or stress-induced cellular molecules. They are localized in the cytoplasm and various organelles where they act as chaperones or proteases. Based on molecular weight, HSP are categorized into several chaperone classes, including Hsp100, Hsp90, Hsp70, Hsp60, Hsp40, and the small HSP (Kampinga et al. 2009). Intracellularly expressed mammalian HSP are widely studied molecular chaperones in essential cellular processes like polypeptide folding and translocation as well as native protein stabilization upon either physiological or pathological conditions. In addition, since various HSP are overexpressed in inflamed tissues and their presence in the extracellular space has been associated with the inflammation process, HSP became the focus of interest of scientists in the context of the autoimmune process. Both bacterial and autologous HSP are highly immunogenic in nature, and the immune response to these proteins has been observed in various inflammatory and autoimmune diseases. For instance, immunoreactivity to Hsp60 chaperone is predominantly aimed at suppression of immune response. This has been confirmed in several murine models of autoimmune arthritis resembling rheumatoid arthritis (RA) and juvenile idiopathic arthritis (JIA) in human. Interestingly, only highly conserved Hsp60-derived epitopes used for animal immunization were disease-regulating in murine model of arthritis and self-Hsp60-reactive T cells displayed anergic phenotype with immunosuppressive potency. Therefore, self-Hsp60 epitopes may serve as altered peptide ligands (APLs) used for therapeutic purpose (Kim et al. 2016; Dominguez et al. 2011). While interactions between bacterial- or self-HSP and host immune system components lead to stimulation of humoral (auto)immune response and the production of anti-HSP, numerous basic and preclinical studies revealed immunosuppressive activity of some HSP (van Eden et al. 2005a, b; Wieten et al. 2009; Stocki and Dickinson 2012; van Herwijnen et al. 2012; Raz et al. 2014; Tukaj 2014; van Eden et al. 2017; Ulmansky et al. 2015). Mechanistically, both bacterial and autologous HSP are able to activate immune regulatory mechanisms, including expansion of T regulatory (Treg) cells and/or T helper 2 (Th2) cell population as well as arrest polarization of pro-inflammatory T helper 1 (Th1) cell population (van Eden et al. 2005a, b; Wieten et al. 2009; van Herwijnen et al. 2012; Stocki and Dickinson 2012; Tukaj 2014; van Eden et al. 2017). Immunosuppressive nature of autologous Hsp60 has also been partly confirmed in patients with JIA, because the disease remission was positively correlated with higher serum levels of Hsp60 and the immunoreactivity of T cells against this chaperone (Kim et al. 2016). Since inadequate activity and/or number of Treg is a characteristic feature of most autoimmune diseases (Long and Buckner 2011), therapeutic strategies which aim to induce immunoregulatory mechanisms and prevent uncontrolled activation of effector cell populations are highly warranted. In this perspective and reflection article, the potential usage of HSP in the context of autoimmune disease therapy is presented.

## Heat shock proteins as molecular chaperones

HSP are a group of constitutive and/or stress-induced proteins present in all known cellular organisms. “Heat shock proteins” is something of a historical term for stress proteins. It seems insufficient because thermal stress is only one of many known stimuli of HSP in the cell. Beside heat, diverse internal and external stresses like cold, ethanol, trace metals, ROS, NO, UV, infection, or inflammation constitute the most common HSP inducers mentioned in the scientific literature. In other words, all stress stimuli which lead to relaxation of physiological structure of proteins followed by exposition of their hydrophobic regions drive to HSP induction. Under physiological conditions, constitutively expressed HSP with chaperone activity participate in protein quality control and help in protein folding in the cell. Newly synthesized polypeptides very often require the assistance of such HSP to efficiently fold into functional three-dimensional native structure. First, ribosome-associated chaperones help in the initial folding steps and additionally protect elongating polypeptide chains from misfolding and aggregation. Later, partly folded polypeptides may require further support from other chaperones or acquire native form spontaneously. In summary, HSP assist in de novo polypeptide folding, refolding of denatured, misfolded or aggregated proteins, assembly of oligomeric proteins, protein transport, proteolytic degradation, and stabilize the structure of native proteins (Ellis 1987; Nollen and Morimoto 2002; Tukaj and Węgrzyn 2016).

## HSP are actively secreted from the cell

There are two basic sources of extracellular HSP in animals and humans, i.e., microbial- (both infectious and commensal) and self-derived HSP. In the past, the presence of bacterial- and self-HSP in the extracellular space was only associated with dying cells and they were regarded as highly immunogenic molecules with pro-inflammatory activity contributing to the development of autoimmunity. Pro-inflammatory activity of HSP, however, has been contested by many authorities because the removal of endotoxin contaminations from these molecules (co-purified from bacterial extracts) revealed their immunosuppressive activity in vitro (Broere et al. 2011; van Eden et al. 2012). While the mechanism of mammalian HSP secretion needs to be further elucidated, there is no doubt that their presence in the extracellular space is due to necrosis or active secretion from the living cells displaying numerous activities, including tissue remodeling via matrix metalloproteinases activation or wound healing via cell motility stimulation (Atalay et al. 2009; de Maio 2011; Sims et al. 2011; Guo et al. 2017). The mechanism of HSP liberation form the eukaryotic cell is still enigmatic, because none of HSP poses secretory signals. Therefore, it seems that the export of HSP to the outside of the cell is conducted by the alternative pathways, including extracellular vesicles (de Maio 2011). In addition, it has been shown that self-HSP are among the most frequent major histocompatibility complex (MHC) ligand sources in antigen presenting cells (APC) (Paludan et al. 2005). Furthermore, the contribution of autologous HSP in the positive selection of T cells in the thymus, including natural T regulatory cell (nTreg) fraction, has been proposed (Adamopoulou et al. 2013; van Eden et al. 2017).

## Immunosuppressive activity of HSP

Inflammation is well-characterized physiological process manifested by the inter alia overexpression of HSP in the cell. Whether upregulation of HSP in chronically inflamed tissues act as “friends” or participate in pathology process remains unclear. On the one hand, pharmacological induction of HSP in the cell may downregulate inflammation via, e.g., activation of heat shock factor 1 (HSF-1) and attenuation of NFκB activity (Wieten et al. 2007, 2010a, b; Tukaj and Węgrzyn 2016). Besides, moving back to the chaperone activity of HSP, it is hard to believe that highly conserved and widely distributed HSP could be directly involved in pathology. On the other hand, however, abundantly expressed HSP in most of cancer cells promote their growth and survival and it has been proved that pharmacological downregulation of HSP expression as well as blockage of their chaperone activity resulted in tumor regression (Ciocca et al. 2012; Li et al. 2013; Tukaj and Węgrzyn 2016). Nevertheless, numerous preclinical studies revealed that active immunization with full-length HSP or their evolutionary conserved peptides suppress inflammation (Wieten et al. 2009; van Herwijnen et al. 2012) and the majority studies regarding anti-inflammatory HSP actions concern highly expressed Hsp60 and Hsp70 chaperones (van Eden et al. 2005a, b, 2017). For instance, immunization with bacterial (*Mycobacterium tuberculosis*) or mammalian Hsp60 ameliorated or suppressed various experimental models of autoimmune diseases, e.g., adjuvant arthritis, collagen-induced arthritis, diabetes type I, or atherosclerosis in a Th2- and/or Treg-dependent way (van Eden et al. 2005a, b; Landstein et al. 2015). Moreover, an altered peptide ligand (APL) derived from a CD4+ T cell epitope of human Hsp60 inhibited the course of murine arthritis, similarly to methotrexate (Lorenzo et al. 2017), and increased the percentage of Treg or downregulated the secretion of pro-inflammatory IL-17 in peripheral blood mononuclear cell cultures from RA patients (Barberá et al. 2016). Further, artificial induction of Hsp70 expression or administration of bacterial or murine Hsp70-derived peptides prevented inflammatory damage in experimental autoimmune arthritis models in a Treg- and/or IL-10-dependent way (van Eden et al. 2005a, b; Stocki and Dickinson 2012; Borges et al. 2012; van Herwijnen et al. 2012; van Eden et al. 2013a, b). In addition, we have recently found that active immunization with Hsp70 attenuated autoimmune-like psoriasis development in a mouse model (Tukaj et al. unpublished observation).

## Anti-HSP (auto)antibodies in autoimmune diseases

It is well established that bacterial- and self-HSP epitopes interact with adaptive arms of the immune cell components (van Eden et al. 2005a, b, 2017). These interactions may stimulate humoral (auto)immune response and the production of anti-HSP (auto)antibodies. Although anti-HSP autoantibodies are present in the sera of healthy individuals (Pockley et al. 1998, 1999) and at elevated levels in patients suffering from various autoimmune diseases, including rheumatoid arthritis (RA) (Tukaj et al. 2010; Mantej et al. 2019), dermatitis herpetiformis (DH) (Kasperkiewicz et al. 2014), coeliac disease (CD) (Tukaj et al. 2017), and autoimmune blistering skin disease like epidermolysis bullosa acquisita (Tukaj et al., unpublished observation), their physiological and pathological role in and value for predicting the development and/or progression of autoimmunity is not completely understood. This is especially intriguing since naturally occurring as well as acquired *Mycobacterium tuberculosis* anti-Hsp60 antibodies protect against the induction of murine model of arthritis (Ulmansky et al. 2002). In addition, humanized anti-Hsp60 mAb (Prozumab) was found to be effective in protecting and suppressing murine models of arthritis and colitis, and the presence of anti-Hsp60 mAb in anti-CD3-stimulated human peripheral blood mononuclear cell (PBMC) cultures downregulated the secretion of pro-inflammatory IL-6 and IFN-ɣ and stimulated anti-inflammatory IL-10 (Ulmansky et al. 2015). In RA patients, we have observed that despite higher serum levels of anti-HSP40 (auto)antibodies, bacterial and human HSP40 inhibited T cell proliferation and stimulated secretion of anti-inflammatory IL-10 in patients’ PBMC cultures (Tukaj et al. 2010). Further, we have recently found that increased levels of autoantibodies to Hsp60, Hsp70, and Hsp90 in RA patients are not associated with the disease progression or activity. Interestingly, positive correlation between serum levels of anti-Hsp60 IgG and IL-4, a cytokine characteristic of Th2 population, and inverse correlation between serum levels of anti-Hsp70 IgM and pro-inflammatory TNF-α have been observed in RA (Mantej et al. 2019). In fact, mentioned above observation has been indirectly proved experimentally in vivo. In murine models of arthritis, for instance, active immunization with bacterial or autologous Hsp70—which obviously generated Hsp70-specific immunoglobulins—prevented and arrested the disease symptoms and attenuated inflammation response via induction of IL-10 and Tregs (Wieten et al. 2009; van Herwijnen et al. 2012). Therefore, it seems that generation of anti-HSP (auto)antibodies in animals and humans is not in contradiction with evidence of HSP contribution in the regulation of immune response. At this moment, we cannot exclude the indirect role of anti-HSP (auto)antibodies in the modulation of immune responses.

## HSP in clinical trials

There are only few clinical observations that show promising effects of HSP therapy in patients with rheumatoid arthritis (RA) and patients with diabetes type I. The former trial concerned highly conserved bacterial Hsp40-derived peptide (dnaJP1) which was orally administered to 15 patients with early RA. The therapy was well-tolerated and led to increased production of regulatory cytokines IL-4 and IL-10 and decreased T cell proliferation or production of pro-inflammatory IL-2, IFN-γ, and TNF-α (Prakken et al. 2004). Later, 160 patients with active RA were enrolled in double-blind, placebo-controlled, pilot phase II trial. The patients received dnaJP1 or placebo orally daily over 6 months. The peptide was safe and well-tolerated by the patients and the therapy led to a significant reduction in the percentage of pro-inflammatory CD4+TNFα+ cell population and a trend toward an increased percentage of CD4+IL-10+ cells and clinical efficacy (Koffeman et al. 2009).

Another study showed that intravenous (i.v.) administration of HSP10 in a randomized, double-blind study in 23 RA patients improved significantly disease activity scores during the 12-week therapy (Vanags et al. 2006).

More recently, BiP, an HSP70 family member, was tested in randomized placebo-controlled, dose ascending double-blind phase I/IIA trial in 24 patients with active RA. Patients received a single i.v. infusion of BiP and the clinical remission was only achieved by patients in the 5- and 15-mg groups, but not patients who received placebo or the lowest dose (1-mg) of BiP (Kirkham et al. 2016). In addition, CIGB 814, an APL from a CD4+ T cell epitope of human Hsp60, an auto-antigen involved in the pathogenesis of RA, has been tested in 20 patients with moderated active RA in an open-label trial. The treatment was well-tolerated and indicated preliminary evidences of clinical efficacy in RA (Corrales et al. 2019).

Another randomized, double-blind, phase I/II clinical trials using immunogenic peptide (p277) from the Hsp60 was performed in recent-onset type 1 diabetes patients (Lazar et al. 2007; Huurman et al. 2008). In these studies, C-peptide-positive patients received subcutaneous injections of p277. Clinical trials proved a significant preservation of β cell function in adult patients compared to placebo-treated patients with no serious adverse immunological side effects. Mechanistically, it has been shown that cytokine production in response to the therapy was dominated by the anti-inflammatory IL-10 (Huurman et al. 2008). The efficacy of this therapy, however, needs to be further investigated, because recently, published optimistic results on treatment of recent-onset type 1 diabetic patients with DiaPep277 in a double-blind, placebo-controlled, randomized phase III trial have been retracted by the authors due to incorrectly analyzed trial data by the external biostatistics firm (Raz et al. 2014).

## Conclusions

There is some evidence that HSP could be considered as components of “vaccine” used to prevent or suppress autoimmune process via induction of T regulatory cells. While the effects of HSP on inflammation and autoimmune processes are mostly based on in vitro and pre-clinical studies, great hopes are being placed in the widespread use of HSP in prevention and/or treatment of some autoimmune diseases in humans. In fact, a few promising clinical observations with regard to HSP in patients with conditions such as rheumatoid arthritis or diabetes type I have been reported. Nevertheless, the question posed in the title of this article remains unanswered, because long-term efficacy and safety, optimal dosage or the use of HSP in combination with current therapy require further research.
